# Building Blood Vessels—One Rho GTPase at a Time

**DOI:** 10.3390/cells8060545

**Published:** 2019-06-06

**Authors:** Haley Rose Barlow, Ondine Cleaver

**Affiliations:** Department of Molecular Biology and Center for Regenerative Science and Medicine, University of Texas Southwestern Medical Center, Dallas, TX 75390, USA; Haley.Barlow@utsouthwestern.edu

**Keywords:** Rho GTPase, endothelial, blood vessel, lumenogenesis, vasculogenesis, angiogenesis, junction, contractility, barrier function, disease

## Abstract

Blood vessels are required for the survival of any organism larger than the oxygen diffusion limit. Blood vessel formation is a tightly regulated event and vessel growth or changes in permeability are linked to a number of diseases. Elucidating the cell biology of endothelial cells (ECs), which are the building blocks of blood vessels, is thus critical to our understanding of vascular biology and to the development of vascular-targeted disease treatments. Small GTPases of the Rho GTPase family are known to regulate several processes critical for EC growth and maintenance. In fact, many of the 21 Rho GTPases in mammals are known to regulate EC junctional remodeling, cell shape changes, and other processes. Rho GTPases are thus an attractive target for disease treatments, as they often have unique functions in specific vascular cell types. In fact, some Rho GTPases are even expressed with relative specificity in diseased vessels. Interestingly, many Rho GTPases are understudied in ECs, despite their known expression in either developing or mature vessels, suggesting an even greater wealth of knowledge yet to be gleaned from these complex signaling pathways. This review aims to provide an overview of Rho GTPase signaling contributions to EC vasculogenesis, angiogenesis, and mature vessel barrier function. A particular emphasis is placed on so-called “alternative” Rho GTPases, as they are largely understudied despite their likely important contributions to EC biology.

## 1. Introduction

Blood vessels are essential for the survival of any organism or tissue larger than the oxygen diffusion limit. Despite their importance, there is much still to learn about their growth and maintenance. How and why does a blood vessel form when and where it does? What rules regulate the diameter of blood vessel lumens? How is the permeability of mature vessels regulated in response to diverse stimuli? How do blood vessels adapt to maintain perfusion of the entire organism during growth or after injury? The answers to these questions are often complicated, as endothelial cells (ECs) that make up the inner lining of all blood vessels grow new vasculature by a wide variety of cellular mechanisms.

During early embryonic development, initial blood vessels form via vasculogenesis, whereby endothelial progenitors called angioblasts migrate together to form rope-like cords, which then coordinately open central lumens [1]. Formation of new vessels later in development and in adulthood relies primarily upon sprouting angiogenesis, in which new vessels sprout from pre-existing ones [2,3]. Blood vessels can also grow by remodeling angiogenesis, including intussusception and anastomosis, where vessels split or fuse respectively [2]. While at a glance the wide array of blood vessel growth mechanisms may seem fundamentally different, the crux of each is the formation (or extension) of an open and continuous lumen, which likely involves conserved cellular processes. Most of these processes involve cellular responses to extracellular Vascular Endothelial Growth Factor (VEGF) and most of them involve complex intracellular signaling pathways still being unraveled.

Our group has long examined the basic cellular behaviors that drive blood vessel formation [2]. To form a functional vessel via any of the above growth mechanisms, ECs must regulate proper cell migration and polarization, junctional maintenance and remodeling, and changes in cell shape regulated by cytoskeletal dynamics [4,5]. Many of these mechanisms are also critical to the maintenance of mature vessels, as EC permeability must be actively regulated to execute the function of mature blood vessels [6]. Understanding the signaling pathways that drive basic cellular processes during blood vessel formation and maintenance is key to developing therapies for any of the many diseases that depend upon blood vessel mis-regulation for their progression, including cancer and ischemic diseases such as heart attack.

### 1.1. GTPases Are Powerful Biomolecular Switches

An ever-growing body of work has demonstrated that Ras homology (Rho) GTPase signaling controls many cellular processes underlying organ and tissue morphogenesis–cell migration, adhesion, shape, and proliferation, just to name a few. More recently, these small membrane-bound molecules have been shown to regulate development, growth, maintenance and disease of blood vessels. There is a vast array of Rho GTPases, with 21 proteins identified in mammals [7]. Rho GTPases are an exciting field of study that is growing rapidly, with regular discovery of novel functions for GTPases and their effectors. Our group and others have shown that many of these GTPases are either expressed or active in remarkably cell type-specific manners. These findings promise a wellspring of discovery still ahead.

**Figure 1:**
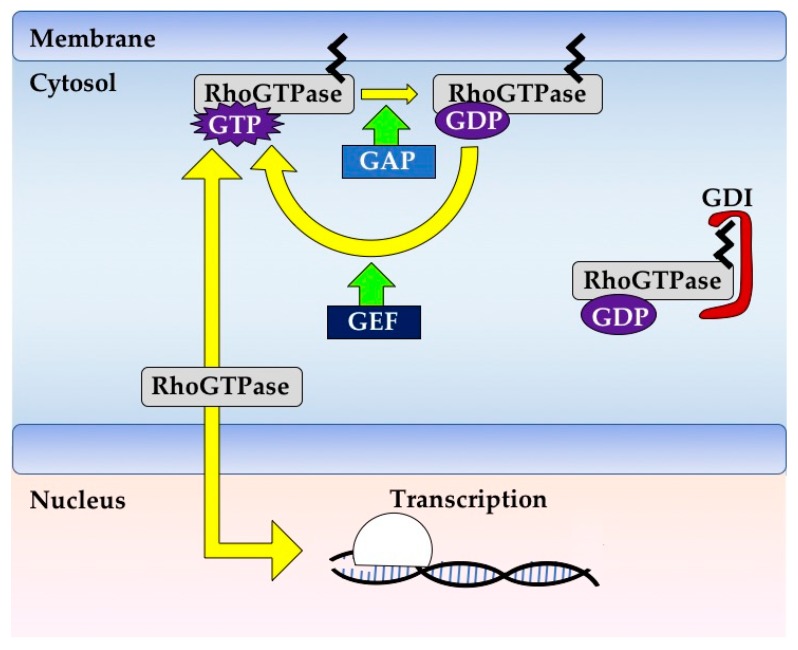
Rho GTPase signaling. Rho GTPases are typically membrane bound due to post-translational modifications such as prenylation. Each Rho GTPase has its own rate of GTP hydrolysis and nucleotide exchange, and these can be increased by GAPs and GEFs, respectively. Rho GTPases are typically active when bound to GTP and in this state can bind effectors to effect changes in cell signaling. Rho GTPases can be negatively regulated by GDIs, which bind and sequester Rho GTPases away from their active subcellular localization and inhibit their reactivation. Rho GTPases can also be regulated transcriptionally or by phosphorylation (not shown), especially those Rho GTPases that have low rates of GTP binding or hydrolysis.

Most Rho GTPases act as molecular switches and carry out their functions by hydrolyzing GTP to GDP within the cell cytoplasm (Figure 1). Each Rho GTPase is regulated by specific Rho GTPase Activating Proteins (GAPs) which stimulate the hydrolysis of GTP, Rho Guanine-nucleotide Exchange Factors (GEFs) that promote the exchange of GDP for GTP, and Rho Guanosine-nucleotide Dissociation Inhibitors (GDIs) which sequester GDP-bound Rho away from its typical active subcellular localization at membranes and inhibit its reactivation [8] (Figure 1). 

This wide array of regulators can interact specifically with a single Rho GTPase or can promiscuously modulate the activity of several different Rho GTPases, lending a level of complexity to disentangling the often crisscrossing signaling cascades in any given process. Efforts to distinguish primary from secondary defects will be an important challenge to tackle as the field progresses, as experimental manipulation of GTPase function often impacts a range of cellular processes, from the cytoskeleton to junctions to cell shape. This will be particularly important in understanding the control of these GTPases during the formation and differentiation of developing tissues, a field which is receiving increasing attention.

### 1.2. GTPase Regulation Is Fine-Tuned and Context-Dependent

Control of GTPase function can occur both at the level of GTPase activity as described above, or at the level of expression. Some Rho GTPases are enriched in ECs (Table 1). A Rho GTPase and its regulators (either a GEF or a GAP) must physically interact, meaning they must be in be in the same subcellular locale. In addition, recent studies have shown that the presence of any given GTPase can be specific to a specific cell type [9]. This combinatorial control offers nearly infinite modification potential for Rho GTPase signaling throughout various cell types and cellular contexts.

An example where tissue-specific control of GTPase transcription controls its downstream roles is the under-studied RhoB, which is a relatively unstable protein that must therefore be regulated at the level of transcription [10]. By contrast, control of subcellular localization can also determine Rho GTPase function. For example, mutations that dissociate RhoA from the plasma membrane block its ability to control cell contractility, and some Rho GTPases such as Rac1 can localize to various subcellular structures such as mitochondria or the nucleus to execute unique functions [11,12,13]. Some Rho GTPases can even be specific to diseased tissue: for example, RhoJ is thought to be enriched in vessels grown in response to angiogenic factors released by tumors [14]. ECs are an exciting and challenging platform to study the different regulatory pathways of Rho GTPases, as they undergo dramatic morphological changes during vessel growth. In addition, inherent EC heterogeneity across vascular beds offers the unique opportunity to study context-dependent roles of Rho GTPases within similar cellular processes.

Fully understanding the signaling mechanisms driving these processes will be critical for the treatment of a wide range of diseases. The ability to manipulate blood vessel growth has the potential to be hugely beneficial for diseases like cancer or ischemic diseases like heart attack. Meanwhile, the ability to control barrier function in mature vessels has sweeping implications for many diseases, as it could facilitate strengthening of healthy vessels in ischemic diseases or promote breakdown of malignant vessels in tumors. It is also known that mis-regulation of vessel stability plays an important role in diseases such as diabetic retinopathy and anaphylaxis, suggesting that opposing these changes in vessel integrity could alleviate disease severity. The study of Rho GTPase signaling in particular presents an incredible opportunity to discover new cell-type specific drug targets, as Rho GTPase signaling is tightly regulated within cells either by cell-type specific expression of the GTPase or its modifiers. A higher-resolution mechanistic understanding of the complex signaling pathways underlying the fundamental behaviors of ECs throughout the lifetime of a blood vessel will advance both therapeutic development and basic science.

## 2. Rho GTPases: A Diverse Family of Molecules

**Figure 2:**
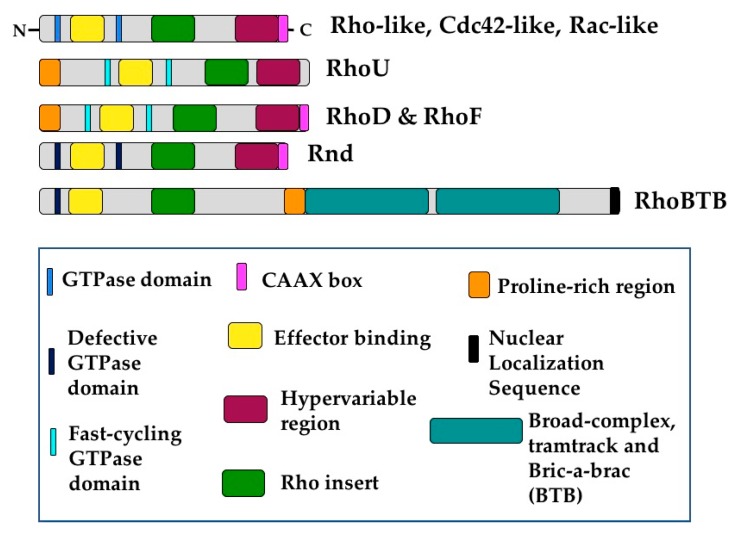
Rho GTPases share conserved structural domains. The essential building blocks of any Rho GTPase are the Rho insert and the GTPase domains. Additionally, Rho GTPases contain effector binding domains and most of them contain C-terminal CAAX domains which can be posttranslationally modified, usually by prenylation. “Classical” Rho GTPases include those of the Rho-like, Rac-like, and Cdc42-like subgroups, as well as RhoU and RhoV, and RhoD and RhoF. RhoU, RhoD, and RhoF deviate from the “classical” formula in that they contain a proline-rich domain at the N-terminus that facilitates binding to other proteins. Additionally, these proteins are considered fast-cycling GTPases, so they are in effect constitutively bound to GTP. Members of the Rnd and RhoBTB subgroups are unable to hydrolyze GTP. Additionally, RhoBTB proteins contain two BTB domains as well as putative nuclear localization sequences.

The 21 known members of the Rho GTPase family can be divided into 8 subgroups (Figure 2) [15]: Cdc42-like (Cdc42, RhoQ (TC10), and RhoJ), the closely related RhoU and RhoV (Chp), Rho-like (RhoA, RhoB, and RhoC), Rac-like (Rac1, Rac2, Rac3, and RhoG), RhoBTB-like (RhoBTB1, RhoBTB2, and RhoBTB3), RhoH, Rnd-like (Rnd1, Rnd2, and Rnd3), and RhoD and RhoF (Rif) [7]. This review will discuss all Rho GTPase family members except RhoV, Rac2, and RhoH, as they are not expressed in ECs during development or in adulthood [16,17,18,19] (Table 1). Additionally, this review does not address the mitochondrial GTPases of the Miro subgroup, as they do not contain Rho inserts and are not widely considered members of the Rho GTPase family [7]. Rho GTPases are considered small GTPases and are grouped together by the conservation of their Rho insert sequences and GTPase domains. A “classical” Rho GTPase contains GTPase domains, a Rho insert, an effector binding domain, and a short C-terminal extension containing a CAAX sequence that can be post-translationally modified, usually by different forms of prenylation (Figure 2) [7,20]. This “classical” Rho GTPase structure is exemplified by those belonging to the Cdc42, Rac, and Rho subgroups. These Rho GTPases primarily act as molecular switches due to their ability to bind and hydrolyze GTP.

Interestingly, this ability is not conserved amongst all Rho GTPases. For example, the non-classical RhoU, RhoD and RhoF undergo GTP hydrolysis and exchange so rapidly that they are essentially constitutively bound to GTP [21]. These proteins also have a N-terminal proline-rich domain, which can facilitate binding to proteins containing SH3 domains [22]. RhoU also varies structurally from the basic Rho GTPase formula as it has extended sequences on both the N- and C-terminal sides of its GTPase domain that alter its activity and subcellular localization [21]. Furthermore, RhoU is typically palmitoylated rather than prenylated, as it lacks the classical C-terminal CAAX domain [23].

The structure of RhoBTB proteins also diverges from that of classical Rho GTPases. The GTPase domains of these proteins are not well conserved, impairing or completely blocking their ability to bind or cycle between GDP and GTP [24,25]. However, the most dramatic variance between RhoBTBs and the more classical Rho GTPases is their increased size. RhoBTB proteins contain a proline-rich domain as well as duplicate BTB (Broad complex, Tramtrack and Bric-a-brac) domains at the C-terminus. Additionally, RhoBTB1 and RhoBTB2 lack a CAAX sequence and are therefore not prenylated (Figure 2) [7,24]. Furthermore, RhoBTB3 hydrolyzes ATP rather than GTP [26], and therefore some hold that it should not be considered a Rho GTPase [7]. Perhaps due to these different domains, RhoBTB proteins have been uniquely associated with proteasomal degradation, acting as adaptors for cullin3-dependent ubiquitin ligase complexes [24,27]. RhoBTB proteins are woefully understudied, especially in EC biology. Given their unique divergences from classical Rho GTPases, it is likely they play exciting and yet undiscovered roles in diverse cellular processes.

Members of the Rnd subfamily are structurally more similar to classical Rho GTPases, containing the Rho GTPase domain with short extensions on either end. Rnd proteins differ most from other Rho GTPases in that they do not seem to affect cellular processes via GTP hydrolysis. Rnd1 and Rnd3 are unable to hydrolyze GTP and therefore constitutively bind GTP, while Rnd2 has very low affinity for GTP or GDP [7,28,29]. This renders these proteins constitutively active, and therefore they must be regulated by other mechanisms such as transcription, subcellular localization, and phosphorylation [30]. These proteins thus likely regulate cell biological processes very differently to their family members. In fact, Rnd proteins have often been shown to directly oppose signaling by classical Rho GTPases, such as RhoA, by binding and inactivating effector proteins [31].

The wide variability in structure and function within this single protein family is extraordinary, and enticingly suggests an abundance of novel signaling mechanisms. Additionally, the structural and sequence similarities between members of subgroups in which primarily one protein has been studied, such as the Cdc42, Rac, or Rho subgroups, hints that the studied roles of these representative proteins may not be as specific as proposed. This demands further study of these lesser-known family members, as they are likely to fine-tune cell biological processes such as migration, proliferation, and changes in cell shape in ways we do not yet understand.

This review aims to summarize the reported roles of Rho GTPases in ECs and to explore the “alternative” Rho GTPases, which lie outside the realm of the well-studied Cdc42, RhoA, and Rac1. Several of these alternative Rho GTPases are expressed at high levels in ECs, and they each have the ability to function differently in ECs and localize to different subcellular structures [32]. The phenotypes of available KO mice for each Rho GTPase are summarized in Table 2, with particular emphasis on EC-specific deletions or phenotypes. Of note, Rnd1 is highly expressed in ECs during development. To our knowledge, no KO mouse exists for its study in ECs, despite data suggesting it is required for angiogenic sprouting from an aortic ring assay [33]. In total, 18 of the 21 Rho GTPases are expressed in ECs during either murine development or in adult vessels, and some of them have not been studied in ECs (Table 1) [16,19]. Understanding how Rho GTPases cooperate to regulate blood vessel growth and maintenance is critical not only to our understanding of cell biology, but also to the advancement of medicine, as discussed above.

## 3. In Vitro Models: The Foundations of Rho GTPase Biology

Much of what is known about the basic functions of Rho GTPases has been characterized using in vitro models, as they allow for easy biochemical and genetic manipulation of ECs [52]. Interestingly, many in vitro models of EC tube formation and angiogenesis faithfully recapitulate vasculogenesis, as this blood vessel formation mechanism occurs by EC coalescence rather than sprouting from existing vessels [52]. In many in vitro assays, a homogenous or heterogeneous mixture of ECs is plated onto a matrix (e.g., collagen, Matrigel, fibroblast-secreted, or fibrin) either with or without other support cells (e.g., fibroblasts or pericytes). Cells are then allowed to adhere and migrate, ultimately forming tubes and/or invading the matrix [53]. In these systems, ECs form luminated vessels, either as individual cells or upon aggregation of groups of cells and cord formation. Cellular behaviors that coordinate to form vessels in these assays include migration, adhesion, polarization, as well as modulation of cell shape and contractility. Indeed, these processes are all required for both vasculogenesis and angiogenesis, making in vitro vascular tubulogenesis assays excellent models for uncovering fundamental Rho GTPase function in ECs to guide in vivo experimentation.

In vitro vasculogenesis models offer many advantages. These assays can allow assessment of large numbers of factors, in a high throughout and highly reproducible platform. These in vitro assays are reductionist assessments of blood vessel formation, and they recapitulate important subsets of in vivo conditions, including EC migration, cord formation and lumenogenesis—all key aspects of vasculogenesis. It is worth noting, however, that some important conditions are missing, including endothelial heterogeneity (vessel size or EC type) and hemodynamics, both normally inherent to tissue vascular beds in vivo. Therefore, it is possible that a given protein studied in ECs isolated from large vessels such as Human Umbilical Vascular ECs (HUVECs) may display different roles than in microvascular cells such as Human MicroVascular ECs (HMVECs) due to the inherent heterogeneity between vascular beds [54]. Another key caveat of studying Rho GTPases in any system, especially in vitro, is that bacterial toxins often used to inhibit signaling of a given Rho GTPase often have broad effects [55]. Therefore, while the use of toxins can be an informative first look at the roles of Rho GTPases in a process of interest, it is critical to follow up with targeted experiments. Interestingly, the amenability of in vitro tubulogenesis assays has allowed assessment of tube formation in cell types other than ECs, suggesting that mechanisms identified using these assays may be universal beyond ECs [56]. Overall, it is clear that our understanding of Rho GTPase function has emerged from classical in vitro studies and informed and catalyzed work later carried out in vivo.

### 3.1. RhoJ Signaling

RhoJ is a Rho GTPase belonging to the classical Cdc42 subfamily and is distinguished by its enrichment in ECs [36]. RhoJ supports tube formation in vitro and localizes to and promotes formation of focal adhesions in migrating ECs [57]. Furthermore, genetic ablation of RhoJ and subsequent failure of lumenogenesis is associated with an increase in RhoA activation and a decrease in Rac1 and Cdc42 activity [58]. In agreement with this, another group demonstrated that knockdown of RhoJ in HUVECs blocked tubulogenesis in vitro by increasing Rho-associated protein kinase (ROCK) activity, which is known to be activated by RhoA [59]. However, it is important to note that, while evidence shows that RhoJ negatively regulates RhoA signaling in HUVECs, ROCK can be regulated by several different Rho GTPases. This suggests that RhoA might not be the only Rho GTPase participating in the signaling cascade.

RhoJ itself responds to VEGF signaling in ECs. Findings, however, are inconclusive as to the functional relationship between RhoJ and VEGF. One group found that RhoJ activity increases in response to VEGF in HUVECs [57], while a different group showed that RhoJ is inactivated by VEGF signaling in the same cell type [60]. Differing experimental conditions or techniques could have resulted in these opposing results, for example, if the activity assay used is not specific to RhoJ, it could result in signal contamination and subsequent mis-measuring of RhoJ activity.

### 3.2. Rac1 Signaling

Rac1 is one of the more well-studied Rho GTPases across various cell types. Its specific role in EC was primarily defined in vitro. Bayless and Davis found that Rac1 signaling is required during later stages of vessel development. They showed that expression of a Dominant Negative (DN) Rac1 HUVECs disrupted lumenogenesis. In their 3D collagen matrix in vitro system, tubulogenesis depends upon the accrual of large intracellular vacuoles that then contribute to lumens. In DN Rac1 cells, vacuoles begin to form but later collapse [61]. These findings are supported by another group that knocked down Rac1 and its family member RhoG, blocking lumen formation [62]. In fact, they outline a signaling cascade in which RhoG upregulates Cdc42 activity, which in turn increases Rac1 activity to promote tubulogenesis—all downstream of VEGF. Interestingly, a different study delineated a strikingly similar signaling pathway containing these three proteins, but following a different epistatic order [63]. RhoG instead upregulates Rac1 which then upregulates Cdc42. In both cases, RhoG appears to centrally regulate these well-known Rho GTPases to specifically influence tubule length, suggesting a role in cell shape or in proliferation. Together, these findings outline how Rac1 is essential in controlling vessel development, via its signaling interactions with other GTPases.

### 3.3. Cdc42 Signaling

Classical and well-studied Rho GTPase Cdc42 plays important roles in several cellular processes during in vitro blood vessel growth. Cdc42 was initially shown to be critical for EC vacuole and lumen formation [61]. Expression of either CA or DN Cdc42 inhibits vacuole formation and subsequent lumenogenesis, suggesting the requirement for fine-tuning Cdc42 activity to properly orchestrate lumen formation. Cdc42 activity was also demonstrated to be critical for EC stress fiber formation, downstream of VEGF in HUVECs [64]. Not surprisingly, Cdc42 activity has also been linked to EC movement. For instance, Cdc42-KO cells in embryoid body assays are able to differentiate into ECs, but the ECs fail to form vascular networks in vitro due to defects in directional migration and network assembly [65]. Indeed, another group showed that Cdc42 is required for cell spreading and migration preceding tubulogenesis [63]. These studies provide an increasingly detailed understanding of the role for Cdc42 in blood vessel formation and tubulogenesis.

### 3.4. Rho Subfamily Signaling

The Rho subfamily is perhaps the most well-known group of Rho GTPases. RhoA, RhoB, and RhoC have often been studied as one functional unit in the history of Rho GTPase studies by utilizing bacterial toxins (e.g., *Clostridium difficile (C.diff)* Toxin B) to inhibit the activity of all three proteins simultaneously. Blocking RhoA, RhoB, and RhoC with *C. diff* Toxin B inhibits vacuole accumulation and subsequent lumenogenesis in HUVECs, but another inhibitor of Rho subfamily proteins (C3 transferase) is unable to fully recapitulate this phenotype [61]. This suggests that these toxins, as discussed above, are not always specific for single Rho subfamily proteins. In fact, *C. diff* Toxin B can target Rho, Rac, Cdc42, RhoG, and RhoQ proteins while C3 transferase targets only RhoA, RhoB and RhoC [55]. This suggests that the observed lumenogenesis defects upon *C. diff* Toxin B inhibition in HUVECs are more likely the result of Rac, Cdc42, RhoG or RhoQ signaling. Further genetic studies have revealed more about the specific roles of these GTPases in lumen formation, but there is still more to discover. In particular, a burning question in the field concerns how these Rho GTPases with similar known functions form a signaling network to regulate cellular behavior.

There are several studies that specifically probe the role of RhoA, but very few that investigate RhoB or RhoC specifically. VEGF signaling can stimulate both RhoA and Rac1 activity and membrane recruitment [66,67,68,69]. Silencing RhoA can rescue overly aggressive migration of HUVECs in which a commonly mutated RhoGAP (DLC1) in cancer has been knocked down. However, KD of RhoA cannot rescue tubulogenesis defects in these mutant cells, suggesting that RhoA may be more important to EC migration rather than processes directly required for EC tubulogenesis [70]. However, another group recently reported that KD of RhoA in vascular ECs in vitro blocks tubulogenesis [62]. Non-mutually exclusive roles for RhoA in both of these processes seem possible, as probing RhoA function via KD may not yield specific results since other Rho GTPases may be able to compensate for its absence. Instead, utilizing CA and DN constructs can give more specific information about the role of a particular Rho GTPase in its active and inactive forms. For example, CA RhoA completely blocks vacuole formation required for lumenogenesis, while a DN RhoA has no effect on this process, suggesting that dampening of RhoA activity is needed to permit lumenogenesis [61,62].

Similar to a handful of other Rho GTPases, RhoB is positively regulated by VEGF in HUVECs, in this case at the level of expression. Interestingly, RhoB negatively regulates RhoA to promote EC migration and vessel formation [71]. Additionally, increased transcription of RhoB mediates the increase in stress fiber formation downstream of Rnd3 overexpression in HUVECs, suggesting that RhoB may act as a broad modifier of the activity of other Rho GTPases [72]. Interestingly, there is minimal study of the basic cellular functions of RhoC in ECs in vitro, suggesting yet another field of potential discovery.

## 4. Vasculogenesis In Vivo

**Figure 3:**
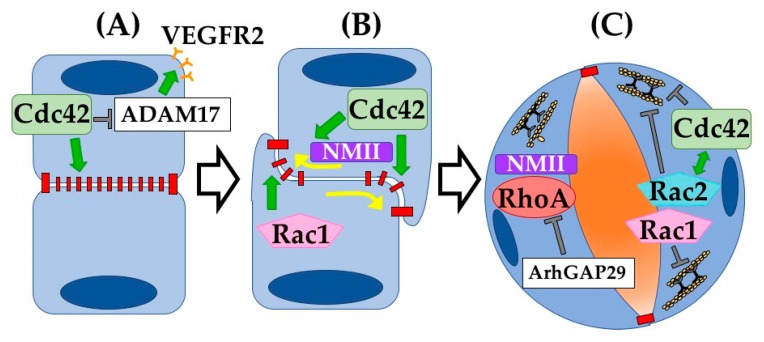
Rho GTPases drive vasculogenesis. Rho GTPases have been shown to be involved in multiple steps during vascular lumenogenesis. (**A**) Cdc42 has been shown to be critical in regulating cytoskeletal organization underlying EC–EC adherens junction assembly. In addition, Cdc42 normally suppresses ADAM17-mediated VEGFR2 shedding. (**B**) Following angioblast cell–cell adhesion, the apical membrane becomes cleared of adherens and tight junctions via Cdc42 activation of NMII-actin contractility. Rac1 functions to build vascular lumens via support of intracellular membrane transport (vacuoles). (**C**) Once the apical membrane forms and the lumen opens, internal contractility of ECs must relax. Our group showed that Arhgap29 suppresses RhoA, and NMII activity, thereby allowing maturing ECs to flatten as the lumen widens. Loss of Arhgap29 or increase in RhoA-NMII activity results in rounding of ECs and narrowing of vessel diameters. Rac1 and Rac2 similarly inhibit EC internal contractility, an essential step towards vascular lumen expansion.

Beyond the in vitro systems discussed above, many insights into the function of GTPases during vasculogenesis have come from in vivo studies. In this work, conditional genetic deletion of GTPases at timepoints prior to vessel formation or to lumenogenesis has revealed their roles in blood vessel development and maintenance. Testing directly in an organism whether and when blood vessels require certain GTPases has underscored their essential nature. Indeed, multiple GTPases were shown to be critical at specific steps of vasculogenesis (Figure 3). 

However, it is important to note the paradoxical difficulty inherent to these studies, when trying to decipher when exactly the targeted gene/protein is successfully deleted and then correctly interpreting the ensuing vascular defects. Given that vessel development generally results from a series of stepwise cellular processes that we are only starting to fully understand, it can be challenging to disentangle the events and their dependence on GTPase function. Secondary effects on neighboring cells resulting from disrupted blood flow and nutrition/oxygenation deprivation inevitably adds to the difficulty in interpreting failures when using in vivo systems. To address this, vascular defects must be assessed quickly prior to cascading abnormalities. Additionally, if a Rho GTPase is not deleted specifically in ECs, it can be difficult to interpret primary defects in ECs from secondary effects in the surrounding tissue. Nonetheless, combining information from both in vitro and in vivo observations helps paint a picture of how and why each GTPase might be required in the vasculature.

### 4.1. Rho GTPases Control Cellular Processes Underlying Vasculogenesis

#### 4.1.1. Cell–Cell Adhesion Formation

Initiating and maintaining cell–cell adhesions is critical for blood vessel formation and mature function. Generating de novo junctions during vasculogenesis requires proper cell polarization and subsequent targeting of junctional proteins to the apical membrane. Cdc42 is a critical regulator of EC polarity and adhesion. Deletion of Cdc42 in ECs during murine development results in embryonic lethality by E10.5 [35,73]. Mechanistically, early deletion (Tie2Cre) of Cdc42 blocks coalescence of angioblasts and lumenogenesis of early vessels. Later deletion (Cdh5Cre^ERT2^) leads to impaired vessel integrity and disorganized F-actin, indicating a failure of cell–cell and cell–ECM adhesion. Intriguingly, different vascular beds displayed different defects in a Cdc42-deletion background depending on the local cellular mechanisms involved: aortic vessels displayed ripping and adhesion discontinuity, cranial capillaries failed to generate lumens, and yolk sac vessels formed large bag-like blood-filled cavities. In vitro, deletion of Cdc42 also reduced filopodia formation and plexus remodeling [35]. Another group similarly utilized Tie2Cre to delete Cdc42 specifically in ECs and observed similar gross defects in both vasculogenesis and angiogenesis. However, they focused primarily on the effect of Cdc42 deletion on VEGFR2 and found that deletion of Cdc42 increased VEGFR2 shedding from the membrane mediated by proteinase ADAM17 [73].

#### 4.1.2. Junctional Remodeling

Once junctions have formed between two ECs, they must be remodeled to facilitate lumen opening. The mechanisms driving this process in ECs are best defined in vasculogenesis. Developing aortae in Cdc42-depleted mice (CAG-Cre^ERT2^) failed to clear junctional proteins from the apical membrane of ECs, resulting in blocked lumenogenesis [35]. Rac1 also plays an important role in junctional remodeling and lumenogenesis. Deletion of Rac1 in ECs (Tie2Cre) caused embryonic lethality by E9.5, suggesting a critical role in early blood vessel growth [39]. Specifically, loss of Rac1 in mouse embryos hampered the development of major vessels and completely blocked the formation of small branched capillaries. Rac1-null aortae, in fact, appear to still be collapsed cords, suggesting defects in lumenogenesis. As discussed above, it can be difficult to tease apart cellular mechanisms in vivo, so Tan et al. turned to an in vitro model to understand the mechanistic defects in Rac1-null ECs. In vitro, this group showed that primary lung ECs are not able to form lamellipodial structures or focal adhesions, nor are they able to remodel their cell–cell contacts, indicating that in vivo lumenogenesis failures may indeed result from defects in junctional remodeling [39].

#### 4.1.3. Cell Contractility and Expansion

Following successful junctional remodeling, ECs are able to open up patent lumens via tight regulation of cell contractility through actomyosin dynamics. In vivo, RhoA activity supports cell contractility via the actin cytoskeleton and needs to be inhibited by ArhGAP29 to facilitate cell stretching and expansion required for lumen opening [74,75]. This finding is confirmed in vitro, as KD of RhoA does not significantly impact early events of lumen formation such as junction remodeling. However, KD of Cdc42, Rac1, or Rac2 all inhibit lumenogenesis. Interestingly, concurrent KD of Cdc42 and Rac2, but not Cdc42 and Rac1, worsens the contractile phenotype thus blocking lumenogenesis, indicating possible cooperation between these signaling molecules in regulation of the actin cytoskeleton [76]. Interestingly, several Rho GTPases are capable of directing changes in the organization of both the actin and the microtubule cytoskeleton in other cell types [77]. Overall, the molecular detail revealed by studying these proteins in vivo exemplifies the intricate complexities of Rho GTPase signaling. Unfortunately, alternative Rho GTPases including those of the Rnd and RhoBTB families are critically understudied in these processes, despite their known roles in vitro and their clear expression in developmental ECs (Table 1). Our understanding of the molecular mechanisms governing lumen formation, this most critical step in blood vessel growth, will benefit greatly from exploration of roles of alternative Rho GTPases.

## 5. Angiogenesis

**Figure 4:**
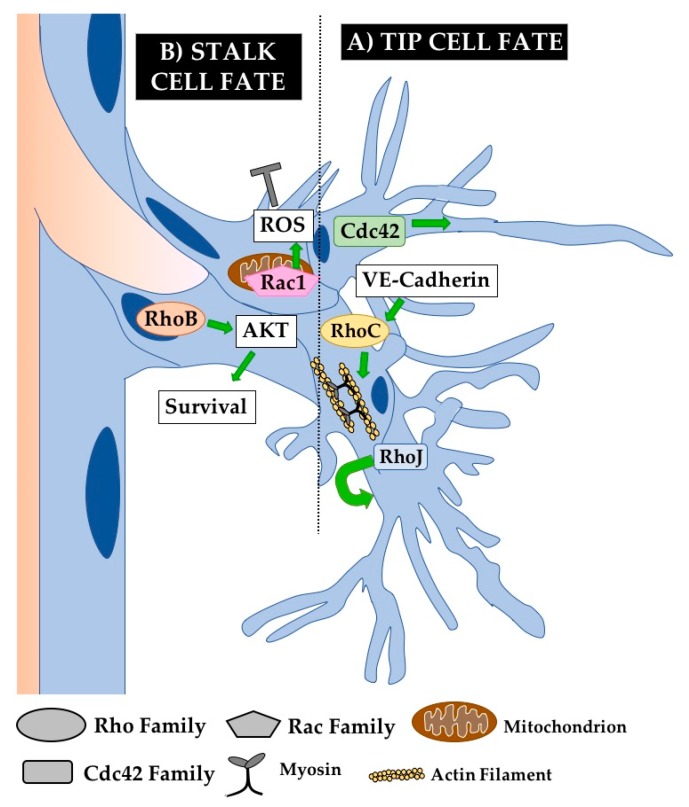
Rho GTPases regulate angiogenesis. Angiogenesis occurs via the formation of sprouts that bud off of pre-existing vessels. Basic components of an angiogenic sprout are the tip cells that explore and follow stimulatory gradients (e.g., VEGF) and the stalk cells that support sprout elongation and lumen extension. (**A**) Cdc42 critically regulates cytoskeletal organization in tip cells to promote the formation of functionally important filopodia. RhoJ also plays an important role in tip cell biology by regulating tip cell selection. (**B**) RhoB is known to regulate stalk cell survival by regulating AKT signaling from the nucleus. Meanwhile, Rac1 regulates the production of reactive oxygen species (ROS) from its unique localization in the mitochondria of stalk cells, inhibiting stalk cell fate. RhoC also plays an important role in regulating actomyosin contractility required for angiogenesis. Not shown are Rho GTPases known to play an important role in angiogenesis but without a defined cellular mechanism, including RhoA and Rac2.

Angiogenesis is the process by which new vessels sprout from existing ones. This is a complex process requiring the coordination of many driving forces including blood flow and cell competition. Angiogenesis is primarily studied in vivo, as it depends on the existence of a pre-existing functional vessel and may also depend on hemodynamic forces. Angiogenesis typically occurs in response to tissue hypoxia, in which hypoxic cells secrete VEGF-A. ECs along existing vessels can respond to this signal via different VEGF-Receptors (typically VEGFR2) and begin sprouting. An angiogenic sprout is made up of tip cells at the leading edge of the growing vessel and stalk cells connecting the tip cell to the parent vessel. The tip cell position is one for which ECs actively compete via Notch signaling—tip cells produce Dll4 in response to VEGF-A and inhibit nearby ECs from becoming filopodia-laden tip cells as well [78]. These filopodia are required for the migratory behavior of tip cells and thus for effective angiogenesis, as cells use these filopodia to explore their surroundings and follow VEGF gradients. Meanwhile, stalk cells proliferate to lengthen the sprout and coordinate new sprout lumenogenesis driven by a combination of autocellular processes and extension of an existing lumen [79]. Studies in the last decade have shown that many of the morphogenetic processes required for angiogenesis including migration, junctional rearrangements, and cell shape changes are governed by Rho GTPases (Figure 4). However as in many other experimental systems, Rho GTPases other than Cdc42, Rac1, and RhoA are relatively understudied in these processes and their investigation comprises an important next frontier for the field.

### 5.1. Rho GTPases Required for Angiogenesis

#### 5.1.1. RhoA Signaling

RhoA has been linked to angiogenesis due to studies of ROCK effector proteins, typically as a VEGFA-responsive factor [80]. Pharmacological inhibition of ROCK strongly inhibits angiogenesis in vivo [81]. However, given that ROCK proteins are regulated by several different Rho GTPases, these findings do not directly address the role of RhoA [81]. RhoA has been specifically shown to play an important role in angiogenesis in vivo by infecting mouse skin with retrovirus-packaged RhoA constructs. Expression of VEGF increases angiogenesis, while co-expression of VEGF and a DN RhoA construct in mouse ECs in vivo blocks new vessel growth. Alternatively, co-expression of VEGF-A and a constitutively active (CA) RhoA significantly increases angiogenesis. However, these new vessels are highly tortuous, suggesting additional roles for RhoA in regulating EC homeostasis or the requirement of closer regulation of RhoA signaling for healthy angiogenesis [82]. It is nonetheless clear that fine balance of RhoA signaling is required for blood vessel formation, which agrees with findings in vitro discussed above and clarifies that RhoA activity indeed promotes angiogenesis in in vivo settings.

#### 5.1.2. RhoB Signaling

While many of the well-studied Rho GTPases are expressed rather ubiquitously, others such as RhoB are more restricted to pathogenic blood vessels. Increased RhoB transcription is associated with the disruptive effects of celiac patient antibodies on angiogenesis [83] and RhoB-null mice have decreased pathological angiogenesis in the ischemic retina, suggesting an important role in disease-specific angiogenesis [46] (Figure 4). Excitingly, an antibody against RhoB decreases angiogenesis in a model of proliferative retina angiogenesis and oxygen-induced retinopathy in an early pre-clinical study, suggesting that RhoB may not be required for vessel maintenance and could be specific to pathogenic vessels [84]. RhoB could represent an exciting new target for disease treatment in which blockage of abnormal angiogenesis, rather than angiogenesis as a whole, is desirable.

#### 5.1.3. RhoJ Signaling

RhoJ is also grossly important for developmental angiogenesis, with KO mice displaying reduced retinal radial growth and an increase in the number of empty sleeves, indicating an increase in vessel regression. However, they did not see a decrease in the number of tip cells, perhaps suggesting a more specific role in stalk cell regulation [36]. Interestingly, overexpression of wild-type RhoJ in ECs (Tie2-Cre) results in a similar phenotype of decreased radial growth of retinal blood vessels. In this case, these angiogenic defects are due to general disruption of actin organization [85] (Figure 4). This indicates a requirement for finely tuned RhoJ activity for proper angiogenesis, as is the case with many of the Rho GTPases that act as molecular switches. RhoJ may carry out this balancing act during angiogenesis by simultaneously inhibiting RhoA activity and upregulating Rac1 and Cdc42 activity [58]. Similar to RhoB above, RhoJ may also have an important role in pathological angiogenesis. RhoJ is enriched in abnormal extraretinal vessels in a model of ischemic retinopathy in mice [60]. Distinguishing the roles of RhoJ in developmental and pathological angiogenesis represents both a great challenge and a great opportunity for discovery, as any marker that may be specific for diseased vessels presents a novel opportunity for therapeutic development.

#### 5.1.4. Rac2 Signaling

Rac2 is also grossly required for angiogenesis, as Rac2 KO aortae are unable to produce angiogenic sprouts in an aortic ring assay. Rac2 KO mice also show defects in the vascularization of the ischemic hindlimb and in invasion of Matrigel plugs. However, Rac2 KO mice are, in fact, viable and fertile, suggesting a role for Rac2 only in angiogenesis in response to damage and not one in normal physiologic processes such as placental development [41]. Although further study is required, Rac2 may also represent a unique target for the treatment of human disease as a regulator of positive EC injury response.

#### 5.1.5. Rnd3 Signaling

Rnd3 has been studied primarily in the context of cardiac angiogenesis following transverse aortic constriction. Whole-body Rnd3^+/-^ mice are viable but with higher incidence of dilated cardiomyopathy and heart failure after transverse aortic constriction. In these mice, angiogenesis following constriction is also impaired, suggesting a role for Rnd3 in angiogenesis after injury, possibly through Hif1α signaling. However, it is not clear whether these effects are primarily cell-autonomous in ECs or non-cell-autonomous, as these mice are full-body knockouts [86]. Even in light of these tantalizing findings, the role of Rnd3 in ECs remains largely unknown. An EC-specific KO in vivo could yield exciting results about how angiogenic vessels form and are maintained by alternative Rho GTPases.

### 5.2. Rho GTPases Control Cellular Processes Underlying Angiogenesis

#### 5.2.1. Tip Cell Defects

Much is known about the role of Cdc42 in tip cell biology, as many of the angiogenic defects in Cdc42-deficient ECs seem to arise from perturbations of tip cell function. Cdc42 promotes the formation of tip cells and filopodia extension in response to extracellular matrix (ECM) polarity cues in zebrafish and in mice, and inhibiting Cdc42 broadly impairs angiogenesis [87] (Figure 4). Another group demonstrated that Cdc42 is required for the development of front-rear EC polarity both in a wound healing assay using primary brain ECs and in response to VEGF-A signaling in angiogenesis in the developing mouse retina [88]. These conclusions are supported by studies utilizing conditional KO of Cdc42 in ECs (Cad5-Cre^ERT2^ or PDGFb-iCre^ERT2^) at P1. Loss of Cdc42 results in broad angiogenic defects in the mouse retina, with a pronounced decrease in the number of branchpoints and total vascular area. While there is not a significant decrease in the overall number of tip cells, there is a clear decrease in the number of filopodia per tip cell [35,89]. These observations are also supported in the study of Cdc42 in the postnatal mouse retina. Here, Cdc42 is required for tip cell selection as well as filopodia formation and directed cell migration, while disposable for EC proliferation or apoptosis and apico-basal polarity [90]. As mentioned above, tip cell filopodia are required for directed angiogenesis [78]. This suggests that Cdc42 activity promotes angiogenesis via cytoskeletal regulation in tip cells, particularly by supporting filopodia generation. RhoJ also regulates tip cell biology, but is instead required for tip cell selection, as KO of RhoJ reduces the number of tip cells in angiogenic tumor vessels in addition to reduced vessel continuity [59]. Together, these findings underscore the fundamental importance of GTPase signaling for tip cell morphology and function.

#### 5.2.2. Sprouting Defects

Despite receiving much less attention in endothelial studies, RhoC has been shown to regulate actomyosin contractility required for angiogenesis [91]. In HUVECs and in zebrafish, Rho-dependent actomyosin contractility is upregulated by VE-cadherin signaling. Interestingly, RhoC expression alone can rescue observed angiogenesis defects in VE-cadherin-inhibited ECs [63].

The similarly under-studied RhoB has also been shown to be required for angiogenesis, both in vitro [92] and in vivo in viable RhoB null mice. In RhoB KO mice, there is decreased pathological angiogenesis in the ischemic retina and in response to cutaneous wounding [46]. To uncover the cell biological role of RhoB, its function has been closely studied in retinal angiogenesis (Figure 4). A closer look at gross angiogenic defects in RhoB null mice reveals delayed development of retinal vasculature with altered sprout morphology. Interestingly, treating neonatal rats with drugs inhibiting the function of RhoB causes apoptosis in sprouting ECs. Knocking down RhoB in primary EC culture also results in EC apoptosis and defective tubulogenesis. This suggests a novel role for RhoB during vascular development as a regulator of cell survival. RhoB carries out this role through a novel localization to the nucleus of ECs where it regulates AKT signaling [11]. Similarly, novel and unexpected subcellular localizations of Rho GTPases have recently begun to be discovered, suggesting a wide field of open discovery in alternative roles for even classical Rho GTPases.

For example, Rac1 was recently shown to localize to a novel subcellular localization to carry out part of its function in angiogenic sprouting. Rac1 can translocate into mitochondria to promote the production of reactive oxygen species (ROS) that subsequently decrease brain angiogenesis, pointing out a novel role for the extremely well-characterized Rac1 outside of directly regulating cytoskeletal dynamics [12]. Other studies have shown that Rac1 is required for proper angiogenic sprouting. EC sprouting stimulated by VEGF in HUVECs is dependent upon Rac1 signaling [63]. Further in vivo studies demonstrated that silencing of Rac1 with siRNA decreased angiogenesis into an in vivo Matrigel plug [93], and closer studies using Cdh5-Cre^ERT2^ in vivo confirmed these findings and indicated a unique and more specific role for Rac1 in vertical blood vessel sprouting in the post-natal retina [40].

## 6. Barrier Function in Mature Vessels

Interestingly, much more is known about Rho GTPase regulation of EC adhesion in mature vessels than in developing vessels. EC-EC adhesion forms what is known as the endothelial barrier, which is critical to water and protein balance between the extravascular and intravascular spaces. This barrier is of direct relevance in many diseases, including diabetic retinopathy, pulmonary edema, ischemic stroke, sepsis, and anaphylaxis. As a consequence, there have been a number of focused studies on the roles of Rho GTPases in junction breakdown, restoration, and stabilization in mature vessels. EC adhesion and barrier function are tightly regulated throughout life to maintain vessel integrity in the face of various stressors. Barrier function is actively modulated to allow vessel leakage that facilitates processes such as immune cell diapedesis. Blood vessel permeability can be stimulated by extrinsic factors such as soluble mediators from migrating blood cells or the surrounding tissue and regulated intrinsically by GTPases and the cytoskeleton.

Tight and adherens junctions between individual ECs determine the stringency of the barrier function of a vessel [94]. Nearest to the lumen, the tight junctions are generally composed of Occludin, Claudin, and Jam proteins while the adherens junctions are made up of Nectins, VE-Cadherin, and PECAM [95]. Changes in permeability are executed by directly modulating the presence of these proteins at the membrane and by altering actomyosin contractility to physically disrupt the interactions of these transmembrane proteins [96]. More is known about the roles of RhoA, Rac1, and Cdc42 than about other members of the Rho GTPase family. However, as previously stated, the methods used to study these proteins are not always specific to a particular member of a Rho GTPase subfamily, suggesting undiscovered regulatory roles for other Rho GTPases.

### 6.1. Barrier Stabilization

**Figure 5:**
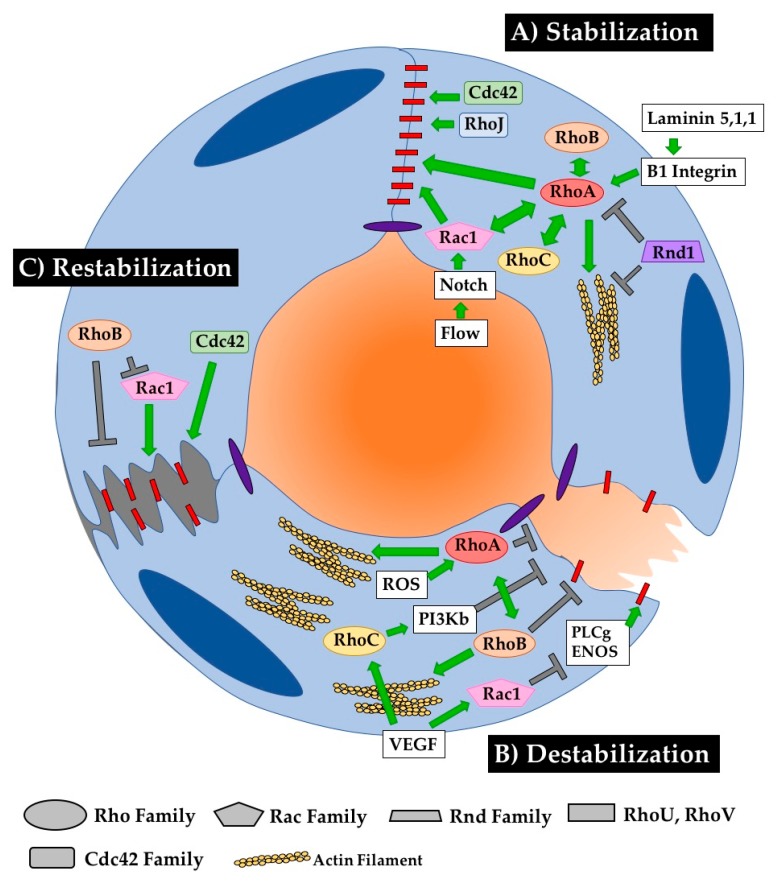
RhoGTPases regulate EC barrier establishment, breakdown and restoration. Close maintenance of blood vessel permeability is critical for the mature function of healthy blood vessels. RhoGTPases play a critical role in stabilizing, destabilizing, and restabilizing the cell–cell connections that dictate vessel barrier function. (**A**) RhoA seems to be the central regulator of stabilizing cell–cell junctions. Several other RhoGTPases, including RhoB, RhoC, Rac1 and Rnd1 modulate barrier function through or with RhoA. RhoJ and Cdc42 also play important roles in stabilizing junctions, as their loss in ECs reduces barrier function. (**B**) The pathways mediating barrier disruption are less streamlined, with RhoA, RhoB, RhoC and Rac1 all playing relatively independent roles in junction disruption. (**C**) Less is known about mechanisms governing barrier recovery, as experimenters do not often carefully distinguish between pathways mediating recovery or proteins that are responding to destabilization of junctions. RhoB inhibits destabilization both by directly targeting junctions and by inhibiting the stabilizing activity of Rac1. Cdc42 also contributes to stabilizing cell–cell contacts after disruption.

Several Rho GTPases play important roles in endothelial junction stabilization, and their signaling must be finely tuned to maintain appropriate barrier function. As depicted in Figure 5, all three Rho proteins are capable of “collaborating” to support HUVEC barrier function [97]. RhoA signaling is increased downstream of β1-integrin signaling, increasing VE-cadherin localization at cell–cell junctions in vitro and thus decreasing permeability across the barrier [98]. RhoA can also preserve barrier function in coordination with Rac1 in corneal ECs [99]. Rac1 is indeed known to regulate assembly of adherens junctions in response to shear stress and changes in cell contractility, establishing barrier function in response to Notch1 signaling [100,101,102]. Tight regulation of Cdc42 signaling is also required for junctional maintenance in mature vessels as it is in newly forming vessels, as conditional deletion of Cdc42 increases inflammatory cell infiltration and vessel leakage upon stimulation with LPS [103]. KO of alternative Rho GTPase Rnd1 leads to RhoA hyperactivation and subsequent impairment of EC barrier function in vitro via the overproduction of contractile stress fibers, indicating a role for Rnd1 in inhibiting RhoA activity to promote normal barrier function [33]. Supporting this conclusion, overexpression of Rnd1 in HUVECs decreases stress fiber formation, which is classically associated with RhoA signaling, to the point of weakening cell–cell junctions [72]. Similarly, KO of RhoJ increases tumor vessel permeability, suggesting a normal requirement for RhoJ in stabilizing vessel junctions [59]. All this emphasizes the importance of finely tuned cooperative signaling balance amongst Rho GTPases to maintain junctional stability in mature ECs. (Figure 5A).

### 6.2. Barrier Destabilization

EC junctions are normally destabilized in response to stimuli such as hypoxia, reactive oxygen species, and/or VEGF [104]. As discussed above, this destabilization can occur through changes in the presence of junctional proteins at the membrane or by changes in cellular tension that physically disrupt transmembrane protein interactions between cells. The typical protein that comes to mind when contemplating this junctional destabilization is RhoA. RhoA is classically thought of as a positive regulator of actin stress fiber formation, which can physically disrupt cell–cell junctions. Indeed, activation of RhoA downstream of reactive oxygen species leads to hyperpermeability in HUVECs [105]. Interestingly, RhoA also coordinates with RhoB in response to hypoxia in human pulmonary ECs to maximize stress fiber formation and actomyosin contractility, thus increasing EC permeability [106]. VEGF signaling can also disrupt cellular junctions via RhoC and by Rac1. Specifically, Rac1 activity increases in response to VEGF signaling by regulating PI3Kβ signaling. Interestingly, a KD of Rac1 in HUVECs can only partially rescue the hyperpermeability phenotype [67]. Enter RhoC, whose activity also increases in response to VEGF signaling in HUVECs and leads to barrier disruption by downregulating phospholipase Cγ-ENOS signaling [91]. It is possible that these two proteins synergize to affect this response to VEGF, although this has not been directly tested. The role of RhoJ has also not been directly tested in cell–cell junction remodeling, although it has been shown to regulate focal adhesion disassembly via the GIT–PIX complex in single migrating HUVECs [37] (Figure 5B).

### 6.3. Barrier Recovery from Damage

Vessel barrier function must be restored following disruption in homeostatic conditions. In vitro, Cdc42 was shown to shift its localization from the cytosol to the membrane halfway through recovery from drug-induced junctional destabilization. This suggests that Cdc42 regulates the re-establishment of AJs to promote junctional stability after damage [107]. Furthermore, the expression of a DN Cdc42 construct in mouse lung vessels inhibited the ability of vessels to recover barrier function following PAR-1 stimulation. Interestingly, another group demonstrated that Cdc42 activation does not change in response to hyperosmotic stress, suggesting that this role in recovery may not be conserved across stress responses [99]. Interestingly, the same group challenges the generally accepted role for RhoA in disrupting barriers. They show that RhoA and Rac1 do not actually induce contraction in hyperosmotically stressed ECs, but instead respond to that stress to restore proper barrier function.

In contrast to the stabilizing role of Cdc42, RhoB inhibits barrier restoration following acute cell contraction upon thrombin stimulation by blocking membrane extension. Interestingly, RhoB colocalizes with Rac1-positive endosomes, inhibiting trafficking of Rac1 to the cell border and thus blocking its function in junctional restoration [97]. This group also shows that RhoB is expressed in inflammatory vessels, including those in inflamed intestines or in hepatic sinusoids that normally have high permeability, suggesting a role for RhoB in responding to inflammation. Indeed, expression of RhoB, RhoA and RhoC increases upon stimulation with inflammatory cytokines such as TNF or IL-1b. All three of these Rho proteins collaborate to sustain barrier function in HUVECs (Figure 5C).

## 7. Rho GTPases in Disease

As demonstrated above, Rho GTPases play a critical role in the growth and maintenance of blood vessels. Not unexpectedly, mis-regulation of these proteins has been linked extensively to a wide range of diseases. Generally, aberrant GTPase activity or expression in blood vessels can lead to vascular malformations during development that manifest later in life or to improper function of blood vessels. The following is a sampling of the known roles of GTPases in disease.

### 7.1. Cerebral Cavernous Malformations (CCMs)

CCMs are large malformations of the vasculature in the brain that can hemorrhage unexpectedly and lead to death [108]. Development of CCMs in mice and in humans has been directly linked to aberrant regulation of RhoA signaling. Upregulation of RhoA protein level and activity is the common downstream change of a knockdown of disease-causing CCM complex genes CCM1, -2, or -3 [109]. In essence, upregulation of RhoA activity leads to increased stress fiber formation and thus decreased junctional integrity, leading to the loss of barrier function and hemorrhaging characteristic of end-stage CCMs [110]. Interestingly, aberrant formation of actin stress fibers in HMVECS is only partially rescued by blocking Rho subfamily proteins with C3 transferase, and is more completely rescued by treatment with a ROCK inhibitor, suggesting that other Rho GTPases could be at play in this phenotype [111]. Furthermore, the same group uses the drug Simvastatin to rescue stress fiber formation in HMVECs, which blocks certain steps of cholesterol synthesis that are required for the prenylation of RhoA as well as many other Rho GTPases. This supports the probability that other Rho GTPases contribute to the formation of CCMs. Indeed, Cdc42 has also been linked to the formation of retinal capillary-venous malformations, as loss of Cdc42 in mouse retina ECs leads to defective axial polarization and cell migration and subsequent vessel malformations [90]. Further detailed study of Rho GTPases in this process will lead to a better understanding of the signaling mechanisms driving this devastating disease.

### 7.2. Complications of Diabetes

The metabolic instability associated with diabetes can fundamentally impair blood vessels [112]. For example, metabolic stress in diabetic mice leads to Rac1 inactivation and subsequent vessel hyperpermeability in Human Aortic ECs (HAECs) [113,114]. Additionally, RhoA is known to play an important role in diabetic retinopathy, as its increased activity in retinal ECs is strongly associated with EC hyperpermeability in models of diabetes [115]. Targeting these proteins could potentially alleviate hyperglycemia-induced blindness in patients by modulating EC permeability.

### 7.3. Anaphylaxis

In mice, RhoA activity increases in response to Histamine, causing an increase in EC permeability. Moreover, EC-specific KO of RhoA in adult mice partially rescues this change in permeability, and inhibiting ROCK can protect against lethal systemic anaphylaxis [45]. This suggests not only a critical role for RhoA in regulating EC permeability in diseases like allergy and anaphylaxis, but also the rescue discrepancy between RhoA KO and ROCK inhibition implies possible roles for other Rho GTPases in this response. Further study of the contributions of these proteins to anaphylaxis could lead to novel life-saving EC-specific treatments.

### 7.4. Tumor Angiogenesis

RhoJ is highly expressed in tumor vasculature and regulates both new vessel growth and formed vessel integrity [59]. Indeed, RhoJ KO mice experience reduced Lewis Lung carcinoma and breast tumor growth and metastasis that is correlated with decreased tumor blood vessel density [37,59]. RhoB expression has also been noted to be higher in tumor vessels compared to adjacent vessels. However, the cell-autonomous role of RhoB has yet to be studied in tumor angiogenesis, as RhoB signaling also functions as a tumor suppressor in breast tumors and therefore its vessel-specific function is difficult to tease apart [116]. More broadly expressed Rho GTPases can also affect tumor angiogenesis. For example, RhoC is essential for VEGF-induced angiogenesis in a study of hepatocellular carcinoma [117]. Similarly, Rac1 in ECs is also responsive to tumor-secreted vasoactive stimuli, resulting in increased vessel permeability and trans-endothelial migration of tumor cells [67]. Rac1 is also associated with angiogenesis, as injection of siRac1 into Neuro2a tumors in mice resulted in almost complete inhibition of tumor growth, correlated with reduced angiogenesis into the tumor [93]. Therapies targeting Rho GTPases have great potential to inhibit angiogenesis-dependent tumor growth, especially when considering the high level of enrichment of some Rho GTPases including RhoB and RhoJ in diseased vessels.

## 8. Conclusions

The last decade has brought significant progress in our understanding of the molecular underpinnings of blood vessel formation and maintenance. However, it is clear that much remains to be discovered, including novel roles for Rho GTPases in ECs. Together, complementary in vitro and in vivo studies have yielded a treasure trove of information regarding how ECs coordinate their shape and adhesion to build and maintain functional blood vessels. These studies have demonstrated that Rho GTPase signaling networks control endothelial cell–cell and cell–ECM adhesion, cell contractility, polarity and barrier function. Coordinating these cellular processes drives the formation and maintenance of new blood vessels. A critical challenge ahead is integrating the growing body of information that consists of increasing numbers of studies accomplished using a variety of methods. Standardizing assays and aligning observations will help normalize and solidify conclusions [118].

Nonetheless, our understanding of the wide range of vascular GTPase functions is growing and gaining sophistication. Their different roles at different times and places, reflects the inherent heterogeneity of blood vessels across different vascular beds. Large versus small vessels, vessels from the brain to the liver, are all likely built and maintained differently, and the differential application of Rho GTPase signaling networks may facilitate these unique roles. In addition, the multiplicity of regulatory mechanisms available to control GTPase activity, from regulating subcellular localization to availability of binding partners, provides nearly endless opportunities for modulation and potential cell-type specific therapeutic targeting. Further study of Rho GTPases will be required to define disease-relevant molecular mechanisms of blood vessel formation and maintenance. The road ahead for further investigating Rho GTPases is as exciting as it is daunting.

## Figures and Tables

**Table 1:** Expression of Rho GTPases in endothelial cells (ECs). Expression of each Rho GTPase during early stages of vasculogenesis (embryonic day (E) 7.5–E8.5) and in adulthood was probed utilizing publicly available single-cell RNA sequencing databases. Of note, RhoV, Rac2 and RhoH are not expressed in ECs neither during early development nor in adulthood. Another important observation is that Rnd1 is well expressed in ECs both during development and in adulthood, however to our knowledge there is no mouse model to facilitate study of its function in vivo.

Rho GTPase	Expressed in ECs between E6.5–E8.5?	Expressed in adult ECs?	Full Body or EC-Specific KO Available?
Cdc42	Yes	Yes	Yes (both)
RhoQ (TC10)	Yes	No	No
RhoJ	Yes	Yes	Yes
RhoU (Wrch-1)	Yes	No	No
RhoV (Chp)	No	No	No
Rac1	Yes	Yes	Yes (both)
Rac2	No	No	Yes
Rac3	Yes	No	Yes
RhoG	Yes	Yes	Yes
RhoBTB1	Yes	Yes	No
RhoBTB2	Yes	Yes	No
RhoBTB3	Yes	No	Yes
RhoH	No	No	Yes
RhoA	Yes	Yes	Yes
RhoB	Yes	Yes	Yes
RhoC	Yes	Yes	Yes
Rnd1	Yes	Yes	No
Rnd2	Yes	No	No
Rnd3 (RhoE)	Yes	Yes	Yes
RhoD	Yes	No	No
RhoF (Rif)	Yes	No	Yes

**Table 2:** Mouse models of Rho GTPase function. Compiled above is a list of Rho GTPases, the mouse models available to study them, and the vascular-associated phenotypes observed in these mice. Where a Cre driver is indicated, researchers utilized a conditional allele (flox/flox construct). KO = knockout, N/A = no available mouse line to our knowledge.

Rho GTPase	Cre Driver	Phenotype	Citation
Cdc42	Full KO	Embryonic lethal E7.5, with obvious defects as early as E5.5	[34]
	Tie2-Cre,	Embryonic lethal by E9–10; angioblast coalescence and lumenogenesis are blocked	[35]
	Cdh5-Cre^ERT2^	Deleted at E11.5—widespread hemorrhaging, failure of EC polarization and lumenogenesis, defects in vessel integrity, actin organization, and cell–ECM adhesion; Deleted from Post-natal day (P) 0–4 —required for angiogenic growth in retina but not for existing vessel stability	[35]
RhoQ	N/A	No information available	
RhoJ	Full KO	Mice viable and fertile; delay in radial growth of retinal vasculature and an increase in empty sleeves	[36]
	Full KO	Mice viable; decrease in tumor angiogenesis	[37]
RhoU	N/A	N/A	
Rac1	Full KO	Embryonic lethal by E9.5	[38]
	Tie2-Cre	Embryonic lethal by E9.5–10.5—improper development of major vessels and lack of small branched vessels	[39]
	Cdh5-Cre^ERT2^	Embryonic deletion (E10.5)—vessel hemorrhaging and decreased vascular area and branch points; Postnatal deletion (P1–P3)—decreased vascular area and branch points, defective angiogenic sprouting, decreased vertical blood vessel sprouting in retina	[40]
Rac2	Full KO	Mice viable and fertile; decrease in sprouting from aortic ring assay, decrease in vascularization of ischemic hindlimb and Matrigel plug assay	[41]
Rac3	Full KO	Mice viable and fertile; ECs not studied	[42]
RhoG	Full KO	Mice viable and fertile; ECs not studied	[43]
RhoBTB1	N/A	N/A	
RhoBTB2	N/A	N/A	
RhoBTB3	Full KO	Some lethality (homozygous weanlings present at 9.2%), mice are viable with reduced size	[44]
RhoA	Cdh5-Cre^ERT2^	Knockout at 4–6 weeks postnatal increases vessel barrier function and prevents passive cutaneous anaphylaxis	[45]
RhoB	Full KO	Mice viable and fertile with reduced size; defective angiogenesis in postnatal retina with tip cells lacking cytoplasmic extensions; decrease in angiogenesis in response to wounding, decrease in pathological angiogenesis in retina after hypoxia	[11,46]
RhoC	Full KO	Mice viable and fertile, ECs not studied	[47]
Rnd1	N/A	N/A	
Rnd2	N/A	N/A	
Rnd3 (RhoE)	Full KO	Heterozygote mice are viable but prone to heart failure after pressure overload and are predisposed to hemodynamic stress; heterozygote mice present dilated cardiomyopathy with heart failure and impaired angiogenesis; one report of full KO causes hydrocephaly; another report of full KO causes embryonic lethality from cardiac arrhythmia	[48,49]
RhoD	N/A	N/A	
RhoF (Rif)	Full KO	Mice viable, no external abnormalities, ECs not studied	[50,51]

## References

[1] Xu K., Cleaver O. (2011). Tubulogenesis during blood vessel formation. Semin. Cell Dev. Biol..

[2] Cleaver O., Krieg P.A., Harvey R.P., Rosenthal N. (1999). Molecular Mechanisms of Vascular Development. Heart Development.

[3] Carmeliet P. (2003). Angiogenesis in health and disease. Nat. Med..

[4] Herbert S.P., Stainier D.Y. (2011). Molecular control of endothelial cell behaviour during blood vessel morphogenesis. Nat. Rev. Mol. Cell Biol..

[5] Drake C.J., Fleming P.A. (2000). Vasculogenesis in the day 6.5 to 9.5 mouse embryo. Blood.

[6] Claesson-Welsh L. (2015). Vascular permeability—The essentials. Upsala J. Med. Sci..

[7] Wennerberg K., Der C.J. (2004). Rho-family GTPases: it’s not only Rac and Rho (and I like it). J. Cell Sci..

[8] Hodge R.G., Ridley A.J. (2016). Regulating Rho GTPases and their regulators. Nat. Rev. Mol. Cell Biol..

[9] Stoeckle C., Geering B., Yousefi S., Rozman S., Andina N., Benarafa C., Simon H.U. (2016). RhoH is a negative regulator of eosinophilopoiesis. Cell Death Differ..

[10] Jahner D., Hunter T. (1991). The ras-related gene rhoB is an immediate-early gene inducible by v-Fps, epidermal growth factor, and platelet-derived growth factor in rat fibroblasts. Mol. Cell. Biol..

[11] Adini I., Rabinovitz I., Sun J.F., Prendergast G.C., Benjamin L.E. (2003). RhoB controls Akt trafficking and stage-specific survival of endothelial cells during vascular development. Genes Dev..

[12] Shi D., Qi M., Zhou L., Li X., Ni L., Li C., Yuan T., Wang Y., Chen Y., Hu C. (2018). Endothelial Mitochondrial Preprotein Translocase Tomm7-Rac1 Signaling Axis Dominates Cerebrovascular Network Homeostasis. Arterioscler. Thromb. Vasc. Biol..

[13] Kranenburg O., Poland M., Gebbink M., Oomen L., Moolenaar W.H. (1997). Dissociation of LPA-induced cytoskeletal contraction from stress fiber formation by differential localization of RhoA. J. Cell Sci..

[14] Masiero M., Simoes F.C., Han H.D., Snell C., Peterkin T., Bridges E., Mangala L.S., Wu S.Y., Pradeep S., Li D. (2013). A core human primary tumor angiogenesis signature identifies the endothelial orphan receptor ELTD1 as a key regulator of angiogenesis. Cancer Cell.

[15] Vega F.M., Ridley A.J. (2008). Rho GTPases in cancer cell biology. FEBS Lett..

[16] Pijuan-Sala B., Griffiths J.A., Guibentif C., Hiscock T.W., Jawaid W., Calero-Nieto F.J., Mulas C., Ibarra-Soria X., Tyser R.C.V., Ho D.L.L. (2019). A single-cell molecular map of mouse gastrulation and early organogenesis. Nature.

[17] Faure S., Fort P. (2015). Atypical RhoV and RhoU GTPases control development of the neural crest. Small GTPases.

[18] Troeger A., Williams D.A. (2013). Hematopoietic-specific Rho GTPases Rac2 and RhoH and human blood disorders. Exp. Cell Res..

[19] Tabula Muris Consortium, Overall Coordination, Logistical Coordination, Organ Collection and Processing, Library Preparation and Sequencing, Computational Data Analysis, Cell Type Annotation, Writing Group, Supplemental Text Writing Group, Principal Investigators (2018). Single-cell transcriptomics of 20 mouse organs creates a Tabula Muris. Nature.

[20] Roberts P.J., Mitin N., Keller P.J., Chenette E.J., Madigan J.P., Currin R.O., Cox A.D., Wilson O., Kirschmeier P., Der C.J. (2008). Rho Family GTPase modification and dependence on CAAX motif-signaled posttranslational modification. J. Biol. Chem..

[21] Shutes A., Berzat A.C., Chenette E.J., Cox A.D., Der C.J. (2006). Biochemical analyses of the Wrch atypical Rho family GTPases. Methods Enzymol.

[22] Kay B.K., Williamson M.P., Sudol M. (2000). The importance of being proline: The interaction of proline-rich motifs in signaling proteins with their cognate domains. FASEB J..

[23] Berzat A.C., Buss J.E., Chenette E.J., Weinbaum C.A., Shutes A., Der C.J., Minden A., Cox A.D. (2005). Transforming activity of the Rho family GTPase, Wrch-1, a Wnt-regulated Cdc42 homolog, is dependent on a novel carboxyl-terminal palmitoylation motif. J. Biol. Chem..

[24] Berthold J., Schenkova K., Ramos S., Miura Y., Furukawa M., Aspenstrom P., Rivero F. (2008). Characterization of RhoBTB-dependent Cul3 ubiquitin ligase complexes—Evidence for an autoregulatory mechanism. Exp. Cell Res..

[25] Chang F.K., Sato N., Kobayashi-Simorowski N., Yoshihara T., Meth J.L., Hamaguchi M. (2006). DBC2 is essential for transporting vesicular stomatitis virus glycoprotein. J. Mol. Biol..

[26] Espinosa E.J., Calero M., Sridevi K., Pfeffer S.R. (2009). RhoBTB3: A Rho GTPase-family ATPase required for endosome to Golgi transport. Cell.

[27] Wilkins A., Ping Q., Carpenter C.L. (2004). RhoBTB2 is a substrate of the mammalian Cul3 ubiquitin ligase complex. Genes Dev..

[28] Nobes C.D., Lauritzen I., Mattei M.G., Paris S., Hall A., Chardin P. (1998). A new member of the Rho family, Rnd1, promotes disassembly of actin filament structures and loss of cell adhesion. J. Cell Biol..

[29] Foster R., Hu K.Q., Lu Y., Nolan K.M., Thissen J., Settleman J. (1996). Identification of a novel human Rho protein with unusual properties: GTPase deficiency and in vivo farnesylation. Mol. Cell. Biol..

[30] Chardin P. (2006). Function and regulation of Rnd proteins. Nat. Rev. Mol. Cell Biol..

[31] Riento K., Guasch R.M., Garg R., Jin B., Ridley A.J. (2003). RhoE binds to ROCK I and inhibits downstream signaling. Mol. Cell. Biol..

[32] Aspenstrom P., Fransson A., Saras J. (2004). Rho GTPases have diverse effects on the organization of the actin filament system. Biochem. J..

[33] Suehiro J., Kanki Y., Makihara C., Schadler K., Miura M., Manabe Y., Aburatani H., Kodama T., Minami T. (2014). Genome-wide approaches reveal functional vascular endothelial growth factor (VEGF)-inducible nuclear factor of activated T cells (NFAT) c1 binding to angiogenesis-related genes in the endothelium. J. Biol. Chem..

[34] Chen F., Ma L., Parrini M.C., Mao X., Lopez M., Wu C., Marks P.W., Davidson L., Kwiatkowski D.J., Kirchhausen T. (2000). Cdc42 is required for PIP(2)-induced actin polymerization and early development but not for cell viability. Curr. Biol..

[35] Barry D.M., Xu K., Meadows S.M., Zheng Y., Norden P.R., Davis G.E., Cleaver O. (2015). Cdc42 is required for cytoskeletal support of endothelial cell adhesion during blood vessel formation in mice. Development.

[36] Takase H., Matsumoto K., Yamadera R., Kubota Y., Otsu A., Suzuki R., Ishitobi H., Mochizuki H., Kojima T., Takano S. (2012). Genome-wide identification of endothelial cell-enriched genes in the mouse embryo. Blood.

[37] Wilson E., Leszczynska K., Poulter N.S., Edelmann F., Salisbury V.A., Noy P.J., Bacon A., Rappoport J.Z., Heath J.K., Bicknell R. (2014). RhoJ interacts with the GIT-PIX complex and regulates focal adhesion disassembly. J. Cell Sci..

[38] Sugihara K., Nakatsuji N., Nakamura K., Nakao K., Hashimoto R., Otani H., Sakagami H., Kondo H., Nozawa S., Aiba A. (1998). Rac1 is required for the formation of three germ layers during gastrulation. Oncogene.

[39] Tan W., Palmby T.R., Gavard J., Amornphimoltham P., Zheng Y., Gutkind J.S. (2008). An essential role for Rac1 in endothelial cell function and vascular development. FASEB J..

[40] Nohata N., Uchida Y., Stratman A.N., Adams R.H., Zheng Y., Weinstein B.M., Mukouyama Y.S., Gutkind J.S. (2016). Temporal-specific roles of Rac1 during vascular development and retinal angiogenesis. Dev. Biol..

[41] De P., Peng Q., Traktuev D.O., Li W., Yoder M.C., March K.L., Durden D.L. (2009). Expression of RAC2 in endothelial cells is required for the postnatal neovascular response. Exp Cell Res.

[42] Corbetta S., Gualdoni S., Albertinazzi C., Paris S., Croci L., Consalez G.G., de Curtis I. (2005). Generation and characterization of Rac3 knockout mice. Mol. Cell. Biol..

[43] Vigorito E., Bell S., Hebeis B.J., Reynolds H., McAdam S., Emson P.C., McKenzie A., Turner M. (2004). Immunological function in mice lacking the Rac-related GTPase RhoG. Mol. Cell. Biol..

[44] Lutz J., Grimm-Gunter E.M., Joshi P., Rivero F. (2014). Expression analysis of mouse Rhobtb3 using a LacZ reporter and preliminary characterization of a knockout strain. Histochem. Cell Biol..

[45] Mikelis C.M., Simaan M., Ando K., Fukuhara S., Sakurai A., Amornphimoltham P., Masedunskas A., Weigert R., Chavakis T., Adams R.H. (2015). RhoA and ROCK mediate histamine-induced vascular leakage and anaphylactic shock. Nat. Commun..

[46] Gerald D., Adini I., Shechter S., Perruzzi C., Varnau J., Hopkins B., Kazerounian S., Kurschat P., Blachon S., Khedkar S. (2013). RhoB controls coordination of adult angiogenesis and lymphangiogenesis following injury by regulating VEZF1-mediated transcription. Nat. Commun..

[47] Hakem A., Sanchez-Sweatman O., You-Ten A., Duncan G., Wakeham A., Khokha R., Mak T.W. (2005). RhoC is dispensable for embryogenesis and tumor initiation but essential for metastasis. Genes Dev..

[48] Yang X., Wang T., Lin X., Yue X., Wang Q., Wang G., Fu Q., Ai X., Chiang D.Y., Miyake C.Y. (2015). Genetic deletion of Rnd3/RhoE results in mouse heart calcium leakage through upregulation of protein kinase A signaling. Circ. Res..

[49] Lin X., Liu B., Yang X., Yue X., Diao L., Wang J., Chang J. (2013). Genetic deletion of Rnd3 results in aqueductal stenosis leading to hydrocephalus through up-regulation of Notch signaling. Proc. Natl. Acad. Sci. USA.

[50] Goggs R., Savage J.S., Mellor H., Poole A.W. (2013). The small GTPase Rif is dispensable for platelet filopodia generation in mice. PLoS ONE.

[51] Kishimoto M., Matsuda T., Yanase S., Katsumi A., Suzuki N., Ikejiri M., Takagi A., Ikawa M., Kojima T., Kunishima S. (2014). Rhof promotes murine marginal zone B cell development. Nagoya J. Med. Sci..

[52] Davis G.E., Black S.M., Bayless K.J. (2000). Capillary morphogenesis during human endothelial cell invasion of three-dimensional collagen matrices. In Vitro Cell. Dev. Biol. Anim..

[53] Staton C.A., Reed M.W., Brown N.J. (2009). A critical analysis of current in vitro and in vivo angiogenesis assays. Int. J. Exp. Pathol..

[54] Viemann D., Goebeler M., Schmid S., Nordhues U., Klimmek K., Sorg C., Roth J. (2006). TNF induces distinct gene expression programs in microvascular and macrovascular human endothelial cells. J. Leukoc. Biol..

[55] Aktories K., Just I. (2005). Clostridial Rho-inhibiting protein toxins. Curr. Top. Microbiol. Immunol..

[56] Donovan D., Brown N.J., Bishop E.T., Lewis C.E. (2001). Comparison of three in vitro human ‘angiogenesis’ assays with capillaries formed in vivo. Angiogenesis.

[57] Kaur S., Leszczynska K., Abraham S., Scarcia M., Hiltbrunner S., Marshall C.J., Mavria G., Bicknell R., Heath V.L. (2011). RhoJ/TCL regulates endothelial motility and tube formation and modulates actomyosin contractility and focal adhesion numbers. Arterioscler. Thromb. Vasc. Biol..

[58] Yuan L., Sacharidou A., Stratman A.N., Le Bras A., Zwiers P.J., Spokes K., Bhasin M., Shih S.C., Nagy J.A., Molema G. (2011). RhoJ is an endothelial cell-restricted Rho GTPase that mediates vascular morphogenesis and is regulated by the transcription factor ERG. Blood.

[59] Kim C., Yang H., Fukushima Y., Saw P.E., Lee J., Park J.S., Park I., Jung J., Kataoka H., Lee D. (2014). Vascular RhoJ is an effective and selective target for tumor angiogenesis and vascular disruption. Cancer Cell.

[60] Fukushima Y., Okada M., Kataoka H., Hirashima M., Yoshida Y., Mann F., Gomi F., Nishida K., Nishikawa S., Uemura A. (2011). Sema3E-PlexinD1 signaling selectively suppresses disoriented angiogenesis in ischemic retinopathy in mice. J. Clin. Investig..

[61] Bayless K.J., Davis G.E. (2002). The Cdc42 and Rac1 GTPases are required for capillary lumen formation in three-dimensional extracellular matrices. J. Cell Sci..

[62] El Atat O., Fakih A., El-Sibai M. (2019). RHOG Activates RAC1 through CDC42 Leading to Tube Formation in Vascular Endothelial Cells. Cells.

[63] Abraham S., Scarcia M., Bagshaw R.D., McMahon K., Grant G., Harvey T., Yeo M., Esteves F.O., Thygesen H.H., Jones P.F. (2015). A Rac/Cdc42 exchange factor complex promotes formation of lateral filopodia and blood vessel lumen morphogenesis. Nat. Commun..

[64] Lamalice L., Houle F., Jourdan G., Huot J. (2004). Phosphorylation of tyrosine 1214 on VEGFR2 is required for VEGF-induced activation of Cdc42 upstream of SAPK2/p38. Oncogene.

[65] Qi Y., Liu J., Wu X., Brakebusch C., Leitges M., Han Y., Corbett S.A., Lowry S.F., Graham A.M., Li S. (2011). Cdc42 controls vascular network assembly through protein kinase Ciota during embryonic vasculogenesis. Arterioscler. Thromb. Vasc. Biol..

[66] Ju L., Zhou Z., Jiang B., Lou Y., Guo X. (2017). Autocrine VEGF and IL-8 Promote Migration via Src/Vav2/Rac1/PAK1 Signaling in Human Umbilical Vein Endothelial Cells. Cell. Physiol. Biochem..

[67] Yao H., Shi W., Wu J., Xu C., Wang J., Shao Y., Wu X., Zhang Z. (2015). Endothelial Rac1 is essential for hematogenous metastasis to the lung. Oncotarget.

[68] van Nieuw Amerongen G.P., Koolwijk P., Versteilen A., van Hinsbergh V.W. (2003). Involvement of RhoA/Rho kinase signaling in VEGF-induced endothelial cell migration and angiogenesis in vitro. Arterioscler. Thromb. Vasc. Biol..

[69] Wu C., Agrawal S., Vasanji A., Drazba J., Sarkaria S., Xie J., Welch C.M., Liu M., Anand-Apte B., Horowitz A. (2011). Rab13-dependent trafficking of RhoA is required for directional migration and angiogenesis. J. Biol. Chem..

[70] Shih Y.P., Yuan S.Y., Lo S.H. (2017). Down-regulation of DLC1 in endothelial cells compromises the angiogenesis process. Cancer Lett..

[71] Howe G.A., Addison C.L. (2012). RhoB controls endothelial cell morphogenesis in part via negative regulation of RhoA. Vasc. Cell.

[72] Gottesbuhren U., Garg R., Riou P., McColl B., Brayson D., Ridley A.J. (2013). Rnd3 induces stress fibres in endothelial cells through RhoB. Biol. Open.

[73] Jin Y., Liu Y., Lin Q., Li J., Druso J.E., Antonyak M.A., Meininger C.J., Zhang S.L., Dostal D.E., Guan J.L. (2013). Deletion of Cdc42 enhances ADAM17-mediated vascular endothelial growth factor receptor 2 shedding and impairs vascular endothelial cell survival and vasculogenesis. Mol. Cell. Biol..

[74] Barry D.M., Koo Y., Norden P.R., Wylie L.A., Xu K., Wichaidit C., Azizoglu D.B., Zheng Y., Cobb M.H., Davis G.E. (2016). Rasip1-Mediated Rho GTPase Signaling Regulates Blood Vessel Tubulogenesis via Nonmuscle Myosin II. Circ. Res..

[75] Xu K., Sacharidou A., Fu S., Chong D.C., Skaug B., Chen Z.J., Davis G.E., Cleaver O. (2011). Blood vessel tubulogenesis requires Rasip1 regulation of GTPase signaling. Dev. Cell.

[76] Norden P.R., Kim D.J., Barry D.M., Cleaver O.B., Davis G.E. (2016). Cdc42 and k-Ras Control Endothelial Tubulogenesis through Apical Membrane and Cytoskeletal Polarization: Novel Stimulatory Roles for GTPase Effectors, the Small GTPases, Rac2 and Rap1b, and Inhibitory Influence of Arhgap31 and Rasa1. PLoS ONE.

[77] Cardama G.A., Gonzalez N., Maggio J., Menna P.L., Gomez D.E. (2017). Rho GTPases as therapeutic targets in cancer (Review). Int. J. Oncol..

[78] Blanco R., Gerhardt H. (2013). VEGF and Notch in tip and stalk cell selection. Cold Spring Harb. Perspect. Med..

[79] Gerhardt H., Golding M., Fruttiger M., Ruhrberg C., Lundkvist A., Abramsson A., Jeltsch M., Mitchell C., Alitalo K., Shima D. (2003). VEGF guides angiogenic sprouting utilizing endothelial tip cell filopodia. J. Cell Biol..

[80] Bryan B.A., Dennstedt E., Mitchell D.C., Walshe T.E., Noma K., Loureiro R., Saint-Geniez M., Campaigniac J.P., Liao J.K., D’Amore P.A. (2010). RhoA/ROCK signaling is essential for multiple aspects of VEGF-mediated angiogenesis. FASEB J..

[81] Wheeler A.P., Ridley A.J. (2004). Why three Rho proteins? RhoA, RhoB, RhoC, and cell motility. Exp. Cell Res..

[82] Hoang M.V., Whelan M.C., Senger D.R. (2004). Rho activity critically and selectively regulates endothelial cell organization during angiogenesis. Proc. Natl. Acad. Sci. USA.

[83] Martucciello S., Lavric M., Toth B., Korponay-Szabo I., Nadalutti C., Myrsky E., Rauhavirta T., Esposito C., Sulic A.M., Sblattero D. (2012). RhoB is associated with the anti-angiogenic effects of celiac patient transglutaminase 2-targeted autoantibodies. J. Mol. Med..

[84] Almonte-Baldonado R., Bravo-Nuevo A., Gerald D., Benjamin L.E., Prendergast G.C., Laury-Kleintop L.D. (2018). RhoB antibody alters retinal vascularization in models of murine retinopathy. J. Cell. Biochem..

[85] Kusuhara S., Fukushima Y., Fukuhara S., Jakt L.M., Okada M., Shimizu Y., Hata M., Nishida K., Negi A., Hirashima M. (2012). Arhgef15 promotes retinal angiogenesis by mediating VEGF-induced Cdc42 activation and potentiating RhoJ inactivation in endothelial cells. PLoS ONE.

[86] Yue X., Lin X., Yang T., Yang X., Yi X., Jiang X., Li X., Li T., Guo J., Dai Y. (2016). Rnd3/RhoE Modulates Hypoxia-Inducible Factor 1alpha/Vascular Endothelial Growth Factor Signaling by Stabilizing Hypoxia-Inducible Factor 1alpha and Regulates Responsive Cardiac Angiogenesis. Hypertension.

[87] Fantin A., Lampropoulou A., Gestri G., Raimondi C., Senatore V., Zachary I., Ruhrberg C. (2015). NRP1 Regulates CDC42 Activation to Promote Filopodia Formation in Endothelial Tip Cells. Cell Rep..

[88] Dubrac A., Genet G., Ola R., Zhang F., Pibouin-Fragner L., Han J., Zhang J., Thomas J.L., Chedotal A., Schwartz M.A. (2016). Targeting NCK-Mediated Endothelial Cell Front-Rear Polarity Inhibits Neovascularization. Circulation.

[89] Sakabe M., Fan J., Odaka Y., Liu N., Hassan A., Duan X., Stump P., Byerly L., Donaldson M., Hao J. (2017). YAP/TAZ-CDC42 signaling regulates vascular tip cell migration. Proc. Natl. Acad. Sci. USA.

[90] Lavina B., Castro M., Niaudet C., Cruys B., Alvarez-Aznar A., Carmeliet P., Bentley K., Brakebusch C., Betsholtz C., Gaengel K. (2018). Defective endothelial cell migration in the absence of Cdc42 leads to capillary-venous malformations. Development.

[91] Hoeppner L.H., Sinha S., Wang Y., Bhattacharya R., Dutta S., Gong X., Bedell V.M., Suresh S., Chun C., Ramchandran R. (2015). RhoC maintains vascular homeostasis by regulating VEGF-induced signaling in endothelial cells. J. Cell Sci..

[92] Sabatel C., Malvaux L., Bovy N., Deroanne C., Lambert V., Gonzalez M.L., Colige A., Rakic J.M., Noel A., Martial J.A. (2011). MicroRNA-21 exhibits antiangiogenic function by targeting RhoB expression in endothelial cells. PLoS ONE.

[93] Vader P., van der Meel R., Symons M.H., Fens M.H., Pieters E., Wilschut K.J., Storm G., Jarzabek M., Gallagher W.M., Schiffelers R.M. (2011). Examining the role of Rac1 in tumor angiogenesis and growth: A clinically relevant RNAi-mediated approach. Angiogenesis.

[94] Wallez Y., Huber P. (2008). Endothelial adherens and tight junctions in vascular homeostasis, inflammation and angiogenesis. Biochim. Biophys. Acta.

[95] Cerutti C., Ridley A.J. (2017). Endothelial cell-cell adhesion and signaling. Exp. Cell Res..

[96] Yuan S.Y., Rigor R.R. (2010). Regulation of Endothelial Barrier Function.

[97] Marcos-Ramiro B., Garcia-Weber D., Barroso S., Feito J., Ortega M.C., Cernuda-Morollon E., Reglero-Real N., Fernandez-Martin L., Duran M.C., Alonso M.A. (2016). RhoB controls endothelial barrier recovery by inhibiting Rac1 trafficking to the cell border. J. Cell Biol..

[98] Song J., Zhang X., Buscher K., Wang Y., Wang H., Di Russo J., Li L., Lutke-Enking S., Zarbock A., Stadtmann A. (2017). Endothelial Basement Membrane Laminin 511 Contributes to Endothelial Junctional Tightness and Thereby Inhibits Leukocyte Transmigration. Cell Rep..

[99] Ortega M.C., Santander-Garcia D., Marcos-Ramiro B., Barroso S., Cox S., Jimenez-Alfaro I., Millan J. (2016). Activation of Rac1 and RhoA Preserve Corneal Endothelial Barrier Function. Investig. Ophthalmol. Vis. Sci..

[100] Polacheck W.J., Kutys M.L., Yang J., Eyckmans J., Wu Y., Vasavada H., Hirschi K.K., Chen C.S. (2017). A non-canonical Notch complex regulates adherens junctions and vascular barrier function. Nature.

[101] Daneshjou N., Sieracki N., van Nieuw Amerongen G.P., Conway D.E., Schwartz M.A., Komarova Y.A., Malik A.B. (2015). Rac1 functions as a reversible tension modulator to stabilize VE-cadherin trans-interaction. J. Cell Biol..

[102] Timmerman I., Heemskerk N., Kroon J., Schaefer A., van Rijssel J., Hoogenboezem M., van Unen J., Goedhart J., Gadella T.W., Yin T. (2015). A local VE-cadherin and Trio-based signaling complex stabilizes endothelial junctions through Rac1. J. Cell Sci..

[103] Lv J., Zeng J., Guo F., Li Y., Xu M., Cheng Y., Zhang L., Cai S., Chen Y., Zheng Y. (2018). Endothelial Cdc42 deficiency impairs endothelial regeneration and vascular repair after inflammatory vascular injury. Respir. Res..

[104] Engelhardt S., Al-Ahmad A.J., Gassmann M., Ogunshola O.O. (2014). Hypoxia selectively disrupts brain microvascular endothelial tight junction complexes through a hypoxia-inducible factor-1 (HIF-1) dependent mechanism. J. Cell. Physiol..

[105] Li X., Liu J., Chen B., Fan L. (2018). A Positive Feedback Loop of Profilin-1 and RhoA/ROCK1 Promotes Endothelial Dysfunction and Oxidative Stress. Oxid. Med. Cell. Longev..

[106] Wojciak-Stothard B., Zhao L., Oliver E., Dubois O., Wu Y., Kardassis D., Vasilaki E., Huang M., Mitchell J.A., Harrington L.S. (2012). Role of RhoB in the regulation of pulmonary endothelial and smooth muscle cell responses to hypoxia. Circ. Res..

[107] Kouklis P., Konstantoulaki M., Vogel S., Broman M., Malik A.B. (2004). Cdc42 regulates the restoration of endothelial barrier function. Circ. Res..

[108] Ene C., Kaul A., Kim L. (2017). Natural history of cerebral cavernous malformations. Handb. Clin. Neurol..

[109] Richardson B.T., Dibble C.F., Borikova A.L., Johnson G.L. (2013). Cerebral cavernous malformation is a vascular disease associated with activated RhoA signaling. Biol. Chem..

[110] Stockton R.A., Shenkar R., Awad I.A., Ginsberg M.H. (2010). Cerebral cavernous malformations proteins inhibit Rho kinase to stabilize vascular integrity. J. Exp. Med..

[111] Whitehead K.J., Chan A.C., Navankasattusas S., Koh W., London N.R., Ling J., Mayo A.H., Drakos S.G., Jones C.A., Zhu W. (2009). The cerebral cavernous malformation signaling pathway promotes vascular integrity via Rho GTPases. Nat. Med..

[112] Shi Y., Vanhoutte P.M. (2017). Macro- and microvascular endothelial dysfunction in diabetes. J. Diabetes.

[113] Han J., Weisbrod R.M., Shao D., Watanabe Y., Yin X., Bachschmid M.M., Seta F., Janssen-Heininger Y.M.W., Matsui R., Zang M. (2016). The redox mechanism for vascular barrier dysfunction associated with metabolic disorders: Glutathionylation of Rac1 in endothelial cells. Redox Biol..

[114] Bruder-Nascimento T., Callera G.E., Montezano A.C., He Y., Antunes T.T., Nguyen Dinh Cat A., Tostes R.C., Touyz R.M. (2015). Vascular injury in diabetic db/db mice is ameliorated by atorvastatin: Role of Rac1/2-sensitive Nox-dependent pathways. Clin. Sci..

[115] Lu Q., Lu L., Chen W., Chen H., Xu X., Zheng Z. (2015). RhoA/mDia-1/profilin-1 signaling targets microvascular endothelial dysfunction in diabetic retinopathy. Graefes Arch. Clin. Exp. Ophthalmol..

[116] Kazerounian S., Gerald D., Huang M., Chin Y.R., Udayakumar D., Zheng N., O’Donnell R.K., Perruzzi C., Mangiante L., Pourat J. (2013). RhoB differentially controls Akt function in tumor cells and stromal endothelial cells during breast tumorigenesis. Cancer Res..

[117] Wang W., Wu F., Fang F., Tao Y., Yang L. (2008). RhoC is essential for angiogenesis induced by hepatocellular carcinoma cells via regulation of endothelial cell organization. Cancer Sci..

[118] Nowak-Sliwinska P., Alitalo K., Allen E., Anisimov A., Aplin A.C., Auerbach R., Augustin H.G., Bates D.O., van Beijnum J.R., Bender R.H.F. (2018). Consensus guidelines for the use and interpretation of angiogenesis assays. Angiogenesis.

